# The role of virulence genes in *Campylobacter* pathogenicity: a perspective from the Gulf Cooperation Council countries

**DOI:** 10.3389/fcimb.2025.1584835

**Published:** 2025-04-25

**Authors:** Mohamed-Yousif Ibrahim Mohamed, Hazim O. Khalifa, Ihab Habib

**Affiliations:** ^1^ Department of Veterinary Medicine, College of Agriculture and Veterinary Medicine, United Arab of Emirates University, Al Ain, United Arab Emirates; ^2^ ASPIRE Research Institute for Food Security in the Drylands (ARIFSID), United Arab Emirates University, Al Ain, United Arab Emirates

**Keywords:** *Campylobacter*, virulence genes, foodborne infection, one health, Gulf Cooperation Council countries

## Abstract

*Campylobacter* spp., primarily *C. jejuni* and *C. coli*, are leading causes of bacterial gastroenteritis worldwide. This review provides an overview of literature on the prevalence and distribution of virulence genes in *C. jejuni* and *C. coli* isolated from both food samples and humans across GCC countries. The reviewed evidence highlights a gap in our understanding of how differences in the virulence profile affect the pathogenicity of *Campylobacter*. Research has shown that *C. coli* is the predominant species found in retail chicken carcasses in the UAE, while *C. jejuni* is more common in chicken carcasses across other GCC countries. Studies also reveal distinct genotypes of *C. jejuni* and *C. coli*, each with varying pathogenicity. These findings underscore the need for further research on the role of virulence genes in shaping the pathogenicity of Campylobacter, which is essential for developing effective intervention and control strategies in the GCC region.

## Introduction

Foodborne pathogens remain an emerging threat to food safety and trade at the international level. Globally in Europe and various other places, foodborne infections are a significant burden to the health of humans (EFSA, 2022). Research from the European Centre for Disease Prevention and Control (ECDC) and the Zoonoses Reports of the European Food Safety Authority (EFSA) shows that cases of campylobacteriosis affecting humans, especially through *C. jejuni* have from time to time in the past decade been higher than those of salmonellosis (Jay-Russell et al., 2013; EFSA, 2019). In the United States of America (USA), the leading microorganisms identified to cause food-borne diseases are *C. jejuni*, *Listeria*, and *Salmonella* (Mohamed et al., 2024, 2025; Mohamed and Habib, 2025). Both Foodborne campylobacteriosis and salmonellosis as monitored by the Foodborne Diseases Active Surveillance Network (FoodNet) of the Centers for Disease Control and Prevention (CDC) have been trending upward in terms of hospitalizations (Tack et al., 2020).

*Campylobacter* spp., microaerophilic bacteria, thrive in oxygen concentrations of 5% to 10%, along with 10% carbon dioxide (El-Naenaeey et al., 2020; Mohamed et al., 2022). Gram negative with a spiral shaped, curved morphology, they are motile rods with flagella, typically existing as commensals in mammal and bird intestines (Martora et al., 2020). Slow growing, they require specialized growth media with charcoal and antibiotics like cephalothin to suppress competing microorganisms in fecal samples. *C. jejuni* and *C. coli*, known for thermotolerance, grow optimally at 42°C (Zhang et al., 2021). However, not all *Campylobacter* spp. share the same thermotolerance; *C. fetus* lacks this characteristic (Ibrahim et al., 2018; Baali et al., 2020). Stool cultures for human diarrhea cases follow standard practices at 42°C, using cephalothin containing media to recover *C. jejuni* and *C. coli*, although this may not suit other species like *C. fetus* or *C. upsaliensis*, which cause illness less frequently (Senok et al., 2007; Tack et al., 2020; Pires and Devleesschauwer, 2021).

The term “virulence genes” generally refers to genes in microorganisms, such as bacteria, that play a role in disease causation and adaptation to stress conditions (Mohamed et al., 2025). These genes are usually related to the pathogenicity of the microorganism or its capacity to exist and create illnesses in the host organism (Mohamed and Habib, 2023). In the case of bacteria such as *Campylobacter* spp., virulence genes that might be affected by virulence stress may be those related to the process through which the bacteria attaches itself to host cells, enters tissues, and avoids being neutralized by the host’s immune system. Additionally, these genes might also be responsible for the microorganism’s acclimatization to stressful conditions including changes in temperature, pH level, and anything else that may be encountered during the infection process or elsewhere outside the host (Lopes et al., 2021). Examination of virulence stress genes is useful for the knowledge of how bacteria become pathogenic and adapt to existence in various settings. Knowledge of such genes helps in understanding the pathogenicity of the microorganism and may be useful in the search for desirable treatments or protective measures against the disease.

Virulence genes such as *cadF* and *flpA* fibronectin-binding proteins help bacteria in uptake to the epithelial cells of the small intestine which is essential for colonization and infection. These proteins help to fix bacteria on the host cell so that they cannot be shed off the gastrointestinal tract (Jakee et al., 2015; Talukdar et al., 2020). *ciaB* belongs to a *Campylobacter* invasion antigen (Cia) protein subfamily that is involved in the invasion of epithelial cells. To breach the intestinal layer and disease as well as circulate in the body invasion is crucial to bacteria (Gharbi et al., 2022). CDT genes of *cdtA*, *cdtB*, and *cdtC* produce subunits that act on the host cell cycle arrest, and apoptosis resulting in cell death and tissue injury that is connected with gastroenteritis manifestations such as diarrhea and inflammation (Nahar and Rashid, 2018). The *flaA* and *flaB* genes are involved in the synthesis of motor flagellin proteins which are required for the bacterial motility to negotiate the mucous layers of GIT for effective colonization and dissemination (Eryildiz et al., 2020). Another stress response gene is *htrA*, a serine protease which involved in the cleavage of the misfolded proteins and proteins that have been exposed to heat shock and oxidative stress within the host to assist bacteria in surviving within the host (Tegtmeyer et al., 2021). The *clpB* gene encodes a molecular chaperone that disaggregates and refolds stress-damaged proteins, aiding in survival under heat and osmotic stress and maintaining various cellular functions during infection (Alam et al., 2021). The *sodB* gene codes for an enzyme that eliminates or reduces oxidation, and enables the bacteria to withstand the attack of reactive oxygen species from the host. Abnormally, the *katA* gene codes for the enzyme catalase that is responsible for the degradation of hydrogen peroxide to water and oxygen which allows bacteria to combat the host’s immune system (Koolman et al., 2015).

Much as the virulence genes such as *cadF* and *ciaB* are responsible for the ability of *Campylobacter* to adhere and invade host cells and induce a strong immune response, they also instigate the necessity of stress response genes such as *htrA* and *sodB* for bacterial survival (Koolman et al., 2015; Nahar and Rashid, 2018). These stress response mechanisms, however, are not only protective of the bacteria and its metabolic processes in stressful conditions, but also toxic to the host since they suppress inflammation in the site of infection that would otherwise hinder bacterial colonization. *Campylobacter* deploys a multitude of proteins and stress response genes which help it to invade the host, avoid the host’s immune responses, and survive within the hostile milieu of the host organism. The site-specific regulation and interdependence of these genes raise the stakes of *Campylobacter*’s pathogenic and survival mechanisms (Talukdar et al., 2020).

In this study, we aim to elucidate background knowledge and recent updates, based on published research, on the virulence genes of *C. jejuni* and *C. coli* in the GCC countries.

## Materials and methods

The study utilized a narrative review of the literature, where each included study serves as the unit of analysis, forming a collective database (Byrne, 2016). Authors extract characteristics from each study, such as the source of *Campylobacter*, identifying *C. jejuni* and *C. coli* as the primary species involved, and virulence factors. To ensure comprehensive coverage, no time frame restrictions were applied to the articles. Searches were conducted on platforms such as PubMed and Google Scholar. Keywords for each country were employed to refine the search “*Campylobacter* virulence genes in Bahrain”.

### Virulence factors in the GCC countries

Campylobacteriosis is considered a significant public health concern, particularly during the summer (Mohamed, 2024). Individuals who work with animals or animal products, such as veterinarians, farmers, poultry processors, slaughterhouse workers, and butchers, face a high risk of infection due to their professions (Habib and Mohamed, 2022). Despite its potential impact on public health, there is a lack of published research on *Campylobacter* infections in humans within the GCC over the last 20 years, highlighting the need for further studies to address this gap.

**Table 1** summarizes the reports of the virulence factors of *C. jejuni* and *C. coli* samples isolated from both foods and humans in GCC countries. One key virulence factor in *Campylobacter* enteritis is the cytolethal distending toxin (CDT), which damages DNA, causing cell cycle arrest and cell death. Inflammation, neutrophil infiltration, and bacteremia suggest invasion as a significant virulence determinant for *Campylobacter* spp (Wooldridge and Ketley, 1997). Our prior research in the United Arab Emirates found the CDT (governed by the CDT A, B, and C operon) in 88.8% of chicken samples from Abu Dhabi supermarkets, suggesting its potential contribution to the invasion process (Habib et al., 2023). In this study we gathered 45 isolates from chicken meats, employing the Whole-Genome Sequencing technique, each *C. coli* isolate identified harbored 7 to 11 virulence factors. Of those factors, some are present in all strains studied and connected with adherence (*cadF* and *jlpA*), colonization, immune avoidance (capsule synthesis and transport, lipooligosaccharide), and invasion (*ciaB*). This research report is the first report that features the characterization of *C. jejuni* and *C. coli* virulence from retail chicken in the UAE. These results prompted us to conduct additional research (Habib et al., 2024) to study *C. jejuni* and *C. coli* prevalence and virulence profiles during the meat processing operations in UAE abattoirs. *Campylobacter* showed high levels of prevalence throughout the entire poultry processing sequence starting from post-defeathering and continuing through evisceration until final chilling. The whole genome sequencing (WGS) showed major distinctions in virulence factor patterns between the two strains of *C. coli* and *C. jejuni*. The virulence factors O-linked protein glycosylation (*pglG*), pseudaminic acid biosynthesis (*pseD* and *pseI*), and flagellin structure (*flaA* and *flaB*) together with cytolethal distending toxin (*cdtA* and *cdtC*) were found at higher rates in *C. jejuni* compared to *C. coli* and this difference reached statistical significance (p < 0.05) through logistic regression analysis. All isolates lacked plasmid-mediated virulence genes and their associated virB-D genes encoding the Type IV secretion system and related *virB* gene clusters were not detected. Similarly, the motility accessory factor (*maf*) family genes responsible for flagellar biosynthesis and phase variation were missing from all examined isolates.

**Table 1:** Prevalence of virulence factors in *C. jejuni* and *C. coli* found in food samples and humans across GCC countries.

Country	Source	Isolates	Virulence Factors on Genomes	References
**Bahrain**	Patients stool from hospital (January 2002 and January 2004)	*C. jejuni* (n = 96)	*cdtB* (52), *Iam* (52), *cdtB* (30), *Iam* (0), *cdtB* (17), *Iam* (10)	(Al-Mahmeed et al., 2006)
**Bahrain and Saudi Arabia**	Chicken (n = 60)	*C. jejuni* (57%) *C. coli* (19%)	ND	(Al Amri et al., 2007)
**Saudi Arabia**	Chicken meat (106)	*C. jejuni* (26.4) *C. Coli* (9.3%)	ND	(Aljasir and Allam, 2025)
	Eggs (*n* = 20)	*Campylobacter* spp. (5%)	ND	(Aziz et al., 2012)
	Chicken producers (*n* = 99)	*C. jejuni* (52%)	ND	(Yehia and Al-Dagal, 2014)
**Qatar**	Chicken meats	*C. jejuni* (n = 146)	*htrB* (91), *cdtB* (90), *clpP* (96), *cadF* (93), *ciaB* (100)	(Abu-Madi et al., 2016)
	Patients stool from Hamad Medical Corporation hospital	*C. jejuni* (n = 174)	*ciaB* (72), *cadF* (60), *cdtB* (87), *htrB* (67), *clpP* (83)	(Aigha, 2014)
**United Arab Emirates**	Chicken meats	*C. coli* (n = 32)	*cadF* (100), *jlpA* (100), *porA* (100), *pebA* (100), *sapA* (0), *pglA* (100), *pglB* (100), *pglC* (100), *pglD* (100), *pglE* (100), *pglF* (100), *pglG* (0), *pglH* (100), *pglI* (93.8), *pglJ* (100), *maf1* (0), *maf3* (93.8), *maf4* (0), *maf6* (0), *maf7* (0), *neuB2* (100), *neuC2* (96.9), *pseA* (100), *pseB* (100), *pseC* (96.9), *pseD* (6.3), *pseE* (34.4), *pseF* (96.9), *pseG* (100), *pseH* (100), *pseI* (40.6), *ptmA* (84.4), *ptmB* (100), *ciaB* (100), *flaA* (0), *flaB* (0), *flaC* (100), *flag* (100), *flgB* (100), *flgC* (100), *flgD* (100), *flagE2* (100), *flgE* (100), *flagG2* (100), *flgG* (100), *flgH* (100), *flgI* (100), *flgK* (100), *flgL* (100), *flgR* (100), *flhA* (100), *flhB* (100), *flhF* (100), *flhG* (100), *fliA* (100), *fliD* (100), *fliE* (100), *fliF* (100), *fliG* (100), *fliH* (100), *fliI* (100), *fliL* (100), *fliM* (100), *fliN* (100), *flip* (100), *fliP* (100), *fliO* (100), *fliR* (100), *fliS* (100), *fliY* (100), *motA* (100), *motB* (100), *pflA* (100), *virB10* (0), *virB4* (0), *virB8* (0), *virB9* (0), *virD4* (0), *aec17* (56.3), *hcp-2* (59.4), *ctdA* (28.1) *cdtB* (90.6), *cdtC* (3.1), *cysC1* (3.1), *ddhA11* (3.1)	(Habib et al., 2024)
		*C. jejuni* (n = 16)	*cadF* (100), *jlpA* (100), *porA* (100), *pebA* (100), *sapA* (0), *pglA* (100), *pglB* (100), *pglC* (100), *pglD* (100), *pglE* (100), *pglF* (100), *pglG* (100), *pglH* (100), *pglI* (93.8), *pglJ* (100), *maf1* (0), *maf3* (100), *maf4* (0), *maf6* (0), *maf7* (0), *neuB2* (100), *neuC2* (100), *pseA* (100), *pseB* (100), *pseC* (100), *pseD* (56.3), *pseE* (100), *pseF* (100), *pseG* (100), *pseH* (100), *pseI* (93.8), *ptmA* (81.3), *ptmB* (81.3), *ciaB* (100), *flaA* (68.8), *flaB* (86.8), *flaC* (100), *flag* (100), *flgB* (100), *flgC* (100), *flgD* (100), *flagE2* (100), *flgE* (100), *flagG2* (100), *flgG* (100), *flgH* (100), *flgI* (100), *flgK* (100), *flgL* (100), *flgR* (100), *flhA* (100), *flhB* (100), *flhF* (100), *flhG* (100), *fliA* (100), *fliD* (100), *fliE* (100), *fliF* (100), *fliG* (100), *fliH* (100), *fliI* (100), *fliL* (100), *fliM* (100), *fliN* (100), *flip* (100), *fliP* (100), *fliO* (100), *fliR* (100), *fliS* (100), *fliY* (100), *motA* (100), *motB* (100), *pflA* (100), *virB10* (0), *virB4* (0), *virB8* (0), *virB9* (0), *virD4* (0), *aec17* (68.8), *hcp-2* (81.3), *ctdA* (100) *cdtB* (100), *cdtC* (100), *cysC1* (0), *ddhA11* (0)	
	Chicken meats	*C. coli* (n = 45)	*cdtA*, *B*, and *C* (89), *cadF* (100), *jlpA* (100), *porA* (93), *pebA* (98), Capsule biosynthesis and transport (100), Lipooligosaccharides (LOS) (100), *ciaB* (100), *virB4* (53)*, B8* (53)*, B9* (53)*, B10* (53)*, D4* (53), Capsule (36), *luxS* (9),	(Habib et al., 2023)

*ND, Not determined.

The study by Abu-Madi, (Abu-Madi et al., 2016) conducted in Qatar focused on *Campylobacter* spp. isolates obtained from supermarkets, including those from Saudi and Qatari producers. The research aimed to assess the presence of virulence stress genes, specifically *htrB*, *cdtB*, *clpP*, *cadF*, and *ciaB*. These findings shed light on the multiple virulence factors associated with adherence, invasion capabilities, and toxin production, all crucial for *Campylobacter* spp.’s ability to colonize and cause disease. The examination of these selected virulence genes in this study holds significant implications for understanding disease pathogenesis. The high prevalence of these genes among the isolates may be attributed to their importance in aiding *C. jejuni* survival in the high temperatures experienced during the summer months in the GCC countries. In another study in Qatar by Aigha (Aigha, 2014), a relatively significant number of *C. jejuni* isolates, showing the presence of the main virulence and stress response genes in isolates collected from Hamad Medical Corporation hospital.

A molecular investigation targeting the CDT (*cdtB*) and invasion-associated marker (*iam*) genes was conducted on *C. jejuni* strains isolated from stool samples obtained between January 2002 and January 2004 in Bahrain. This molecular characterization aimed to establish correlations with socio-demographic and clinical parameters of the patients. Out of the 96 tested *C. jejuni* strains, 50 (52%) exhibited the presence of both *cdtB* and *iam* genes, 30 (31%) showed the *cdtB* gene presence but not the *iam* gene, and 16 (17%) lacked both *cdtB* and *iam* genes. Notably, a significant majority of patients (66 out of 96, constituting 69%) were under the age of 3 years, with markedly higher detection rates of *cdtB*+/*iam*+ and *cdtB*+/*iam*− strains in this particular age group. Strains lacking both virulence genes (*cdtB* and *iam*) led to symptomatic infections in this age group, in stark contrast to older patients who experienced asymptomatic infections when infected with these strains (Al-Mahmeed et al., 2006).

(Senok and Botta, 2009) conducted a study delving into the molecular characterization of *C. jejuni* isolates from Bahrain. Their research presented data that illuminated the link between the presence of combinations of potential virulence genes, the invasive behavior of the bacteria, and the severity of clinical infections.

These studies reflect valuable research work in molecular identification of virulence genes in *C. jejuni* and *C. coli* strains isolated from the GCC countries. They reveal different genotypes in *C. jejuni* and *C. coli* that have different pathogenicity (Senok and Botta, 2009; Aigha, 2014; Habib et al., 2023). Strains positive for *cia*, *iam*, and *cdtB* genes are found to be more invasive, especially in severe infections. The *ciaB* gene is associated with increased invasiveness and the severity of the infection. In strains devoid of all three genes, the *in vitro* invasiveness remains high, which points toward the presence of other unidentified virulence factors (Johansson et al., 2021). *Campylobacter* enteritis affects children below the age of three years (Asuming-Bediako et al., 2019). emphasizing how multiple virulence factors are involved in *Campylobacter* infections in the GCC countries.

## Conclusions

The review demonstrates the intricate virulence mechanisms of *C. jejuni* and *C. coli* in GCC nations with a focus on CDT as the major toxin that causes cellular destruction while triggering inflammation and promoting cell invasiveness. A high occurrence of CDT-associated genes was identified in retail chicken samples from the UAE research which might serve to advance *C. jejuni* and *C. coli* pathogenicity. The virulence determinants found in *C. jejuni* through WGS analysis surpass those of *C. coli* by showing higher numbers of attachment, colonization, and immune-escape factors while displaying distinct flagellar differences glycosylation patterns, and toxin production levels. Research in Qatar and Bahrain establishes that disease severity heavily relies on virulence genes including *cdtB*, *iam*, *ciaB*, *htrB*, and *cadF*. Analyzing *C. jejuni* and *C. coli* isolates from chicken meat reveals significant prevalence rates of these genes which indicates their important position as infection reservoirs for human beings. The pathogenic bacteria are transmitted across different points of the meat supply chain during processing and retail distribution. However, the persistence of invasiveness in strains lacking well-characterized virulence factors indicates the involvement of additional, yet unidentified, pathogenic mechanisms. Collectively, these findings highlight the need for continued molecular surveillance and in-depth investigations into the genetic diversity of *Campylobacter* across the GCC region. Knowledge about virulence mechanisms will help develop specific preventive measures to diminish foodborne *Campylobacter* infections across the GCC.
